# Prominent metallic surface conduction and the singular magnetic response of topological Dirac fermion in three-dimensional topological insulator Bi_1.5_Sb_0.5_Te_1.7_Se_1.3_

**DOI:** 10.1038/s41598-017-05164-9

**Published:** 2017-07-07

**Authors:** Prithwish Dutta, Arnab Pariari, Prabhat Mandal

**Affiliations:** 1 Saha Institute of Nuclear Physics, HBNI, 1/AF Bidhannagar, Calcutta, 700 064 India; 2 Goverment General Degree College, Singur, Hooghly, 712409 India

## Abstract

We report semiconductor to metal-like crossover in the temperature dependence of resistivity (*ρ*) due to the switching of charge transport from bulk to surface channel in three-dimensional topological insulator Bi_1.5_Sb_0.5_Te_1.7_Se_1.3_. Unlike earlier studies, a much sharper drop in *ρ*(*T*) is observed below the crossover temperature due to the dominant surface conduction. Remarkably, the resistivity of the conducting surface channel follows a rarely observable *T* ^2^ dependence at low temperature, as predicted theoretically for a two-dimensional Fermi liquid system. The field dependence of magnetization shows a cusp-like paramagnetic peak in the susceptibility (*χ*) at zero field over the diamagnetic background. The peak is found to be robust against temperature and *χ* decays linearly with the field from its zero-field value. This unique behavior of the *χ* is associated with the spin-momentum locked topological surface state in Bi_1.5_Sb_0.5_Te_1.7_Se_1.3_. The reconstruction of the surface state with time is clearly reflected through the reduction of the peak height with the age of the sample.

## Introduction

Topology protected electronic band structure is of current interest in condensed matter and material science research. Three-dimensional (3D) topological insulator has been established as an important member of this family since the first experimental realization of topology protected surface and bulk state in Bi_1−*x*_Sb_*x*_^1^ and in binary compounds such as Bi_2_Se_3_^2^, Bi_2_Te_3_, Sb_2_Te_3_^3^, etc. This class of ‘insulators’, due to strong spin-orbit coupling, hosts a metallic surface state with unique physical properties^4^. The spin-momentum locked surface state, which is protected by the time-reversal symmetry, makes the electronic transport robust against inelastic backscattering^4, 5^. Theoretical investigations have predicted topological insulators as potential candidates for applications in spintronics, quantum computation, etc^5^. However, the above mentioned topological insulators reported to exhibit high bulk conductivity, instead of a gap between the valence and conduction band^6–9^. It is now well documented that crystal defects - namely antisite defects and vacancies, which introduce large residual carriers, are the main reasons behind such high conductivity of the bulk^9–11^. As a result, the transport response is dominated by the bulk rather than the surface. The misplacement of Se ion from its lattice position causes an increase in negative carrier concentration, which can be as high as ~10^19^ cm^−3^, whereas the antisite Bi and Sb defects produce holes^11^. Thus, by replacing Bi with Sb and Se with Te in proper ratios, one can balance the opposite type of charge carriers and minimize the conductivity. In order to achieve the higher figure of merit, efforts have been made to reduce the carrier density in the bulk by using ternary and quaternary compounds of Bi, Sb, Te and Se^11, 12^. Teramoto and Takayanagi, in their work, reported that high resistivity can be obtained for compounds with chemical formula Bi _2−*x*_Sb_*x*_Te_3−*y*_Se_*y*_ (BSTS) for a certain linear relation between *x* and *y* ^13^. Ren *et al*. reinvestigated the electronic and structural phase diagram to achieve the intrinsic topological insulating state in these compounds and prescribed that the highest resistivity can be realised close to composition Bi_1.5_Sb_0.5_Te_1.7_Se_1.3_^11^. Since then BSTS has been studied by various groups through transport^10, 11, 14–20^ and spectroscopic measurements ^18, 20–24^. Employing terahertz time-domain spectroscopy, Tang *et al*. have shown that the bulk state contribution to transport in Bi_1.5_Sb_0.5_Te_1.8_Se_1.2_ is significantly smaller than the other Bi based topological insulators and comparable to Bi_2_Se_3_ thin films^24^.

Topological insulators are characterised by their symmetry protected novel surface state properties. However, to observe high quality surface state property, one needs to electronically decouple the surface from the bulk. This can be achieved, by making the bulk highly insulating. We have grown high quality single crystals of topological insulator with composition Bi_1.5_Sb_0.5_Te_1.7_Se_1.3_. The bulk resistivity in some of the crystals used in the present study is about an order of magnitude larger than earlier reports^10, 11, 14, 16–20^. Notably, resistivity at low temperature decreases quadratically with temperature which is a clear evidence of Fermi liquid behavior of the surface state. Furthermore, the field dependence of susceptibility (*χ*) exhibits an unusual paramagnetic peak at zero field as an evidence of the spin-momentum locked Dirac cone surface state.

## Results

### Temperature dependence of resistivity for Bi_1.5_Sb_0.5_Te_1.7_Se_1.3_ single crystals

**Figure 1:**
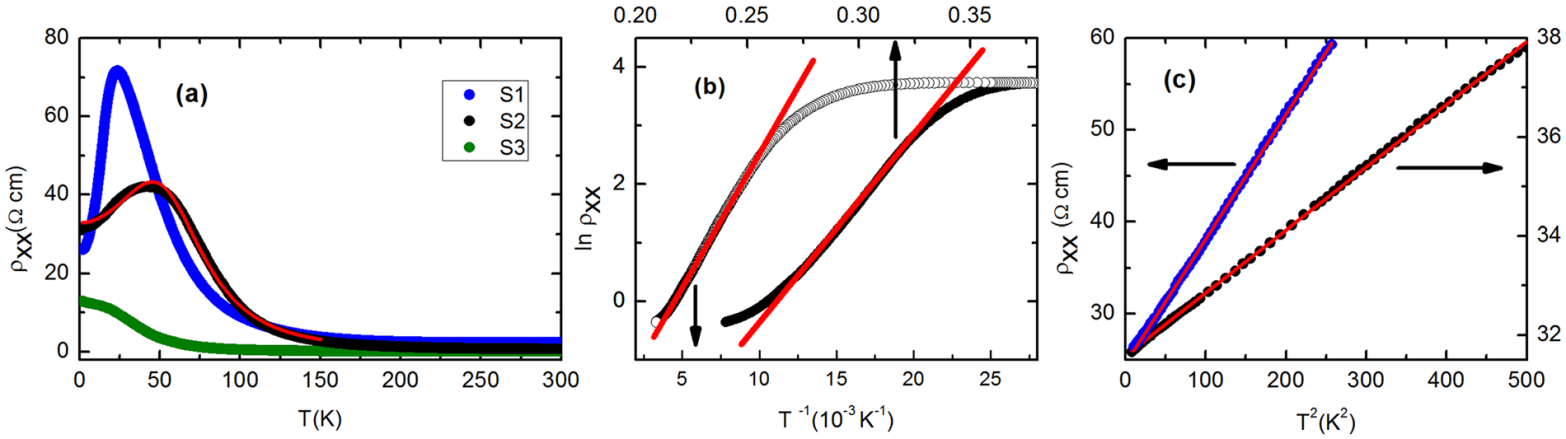
(**a**) Temperature dependence of the resistivity (*ρ*_*xx*_) for Bi_1.5_Sb_0.5_Te_1.7_Se_1.3_ single crystals collected from two different batches. Crystals S1 and S2 were prepared by method II and S3 was prepared by method I. The red line is the fit to the experimental data with equation *ρ*_*xx*_ = [1/*ρ*_*s*_ + 1/*ρ*_*b*_]^−1^, where *ρ*_*s*_( = *a* + *bT* ^2^) and ${\rho }_{b}(={\rho }_{0}\exp {(\frac{{T}_{0}}{T})}^{\mathrm{1/4}})$ are respectively surface and bulk resistivity. (**b**) ln *ρ*_*xx*_ as a function of 1/*T* (open symbol; bottom axis) for the activation behavior and as a function of *T* ^−1/4^ (closed symbol; top axis) for the variable-range-hopping behavior for S2 crystal. (**c**) *ρ*_*xx*_ is plotted against *T* ^2^. The linearity can be traced up to 16 K and 21 K for S1 and S2 crystals respectively, confirming the Fermi liquid behavior of the surface state.

The temperature dependence of resistivity (*ρ*_*xx*_) for three freshly cleaved Bi_1.5_Sb_0.5_Te_1.7_Se_1.3_ single crystals (S1, S2, S3) are presented in Fig. 1(a). Two slightly different techniques were used to prepare these single crystals. Crystal S3 was prepared by method I while crystals S1 and S2 were prepared by method II. The details of preparation are described in the Method section. Phase purity and composition of the crystals were analysed by powder x-ray diffraction and energy dispersive x-ray technique, respectively, which have been shown in Supplementary Figs S1 and S2. With decreasing temperature, resistivity initially increases rapidly and then either decreases or tends to saturate. For example, *ρ*_*xx*_ for sample S2 increases with decreasing temperature and reaches a maximum at ~40 K. Sample S1 also shows similar temperature dependence of *ρ*_*xx*_ with a maximum at slightly different temperature (~23 K). On the other hand, *ρ*_*xx*_(*T*) shows a saturation-like behavior at low temperature for sample S3. The temperature dependence of *ρ*_*xx*_ for sample S3 is similar to earlier reports^10, 11^.

In a usual electrical transport measurement, as the bulk and surface resistance are in parallel configuration, the electrical conductivity (*σ*_*t*_) of the crystal is the sum of surface channel conductivity *G*/*t* and bulk conductivity (*σ*_*b*_), *σ*_*t*_ = *σ*_*b*_ + *G*/*t*, where *t* is the thickness of the sample. However, their relative contribution is sensitive to temperature. Due to the two-dimensional (2D) nature of the surface state, the cross section area of the conducting channel is extremely small. For this reason, the surface resistance can be significantly larger as compared to semiconducting bulk specially at high temperature. Thus, the charge conduction at high temperature is dominated by the bulk. With decrease in temperature, the bulk resistance increases exponentially whereas the surface resistance decreases. This kind of temperature dependence of bulk and surface resistance results in a semiconductor to metal-like crossover at low temperature below which the charge transport is dominated by the surface channel^10, 17–20^. For both technological application and basic research, it is desirable that the bulk conductivity should be very small while the surface conductivity should be high. Several attempts have been made to achieve low bulk conductivity. Most of the reported data on single crystals show that *ρ*_*xx*_ exhibits the expected semiconducting behavior at high temperature. However, at low temperature, *ρ*_*xx*_ becomes either *T*-independent or shows very weak temperature dependence, i.e., the system is barely metallic. To the best of our knowledge, such a strong decrease in total resistivity with decreasing temperature due to metallic surface-state has not been observed so far. Also, the surface conductance ($G\simeq t{\sigma }_{t}=t/{\rho }_{xx}$) at low temperature is expected to show identical behavior, irrespective of the thickness of the sample^15^. The surface conductance, calculated using the above mentioned approximation, are 3.3 × 10^−4^ Ω^−1^ and 3.2 × 10^−4^ Ω^−1^ for S1 (*t* ~ 87 *μ*m) and S2 (*t* ~ 102 *μ*m), respectively. These values are comparable to each other and consistent with the earlier observation^15^.

In order to understand the charge conduction mechanism in Bi_1.5_Sb_0.5_Te_1.7_Se_1.3_, the temperature dependence of resistivity has been analysed. At high temperature, the insulating behavior of *ρ*_*xx*_ can be fitted with the Arrhenius equation, ${\rho }_{xx}={\rho }_{1}\exp (\frac{{\rm{\Delta }}}{{k}_{B}T})$. From the linear behavior of ln *ρ*_*xx*_ versus 1/*T* plot as shown in Fig. 1(b) for sample S2, we have deduced the value of activation energy, Δ ≃ 40 meV. This value of activation energy is comparable to earlier reports^11, 18, 19^. However, in the low-temperature region, *ρ*_*xx*_ cannot be fitted with the above mentioned expression for the activated-type conduction. We have tried to fit the resistivity data with 3D Mott variable-range-hopping equation, ${\rho }_{xx}={\rho }_{0}\exp {(\frac{{T}_{0}}{T})}^{\mathrm{1/4}}$, which has been shown in Fig. 1(b). In this case, a better fitting is obtained with *T*_0_ ~ 5 × 10^6^ K. The observed value of *T*_0_ is quite large. According to the Mott’s variable-range-hopping theory, *T*_0_ is inversely proportional to the density of states [N(E_*F*_)] at the Fermi level and the cube of the localization length (*δ* ^−1^), ${T}_{0}=\frac{16{\delta }^{3}}{{k}_{B}N({E}_{F})}$. The large value of *T*_0_ in Bi_1.5_Sb_0.5_Te_1.7_Se_1.3_ implies that the localization length of the charge carrier in the bulk is very small. *ρ*_*xx*_(*T*) for S1 and S3 samples exhibits qualitative similar behavior. Thus the bulk conductivity shows activation behavior at high temperature but it switches over to 3D variable-range-hopping at low temperature. Crossover from activation to variable-range-hopping has also been reported earlier^11, 19^. In most of the cases, the activation conductivity is observed in a narrow temperature range. In spite of much larger energy gap for some compositions, the bulk resistivity at low temperature is smaller than the present samples^19^. This is possibly due to the smaller value of *T*_0_ (like the present S3 sample) which determines the bulk conductivity at low temperature.

For S1 and S2 crystals, the low-temperature resistivity below the peak, which is dominated by the surface state, shows an upward curvature, suggesting a superlinear temperature dependence of *ρ*_*xx*_ in the metallic region. We observe that *ρ*_*xx*_ below 21 K for sample S2 can be fitted well with the equation *ρ*_*s*_ = *a* + *bT* ^*n*^ for *n* = 2. Figure 1(c) shows *ρ*_*xx*_ versus *T* ^2^ plot. It is clear from the figure that *ρ*_*xx*_ versus *T* ^2^ is strictly linear up to about 21 K. For crystal S1 also, *ρ*_*xx*_ exhibits *T* ^2^ dependence below 16 K. The *T* ^2^ dependence of resistivity at low temperature is the manifestation of Fermi liquid behavior of the surface-state carriers. Electron-electron scattering is known to give rise a *T* ^2^-dependent term in the resistivity. The *T* ^2^ dependence of resistivity is observed in various strongly correlated electron systems such as heavy fermion metals, organic conductors and transition metal oxides. Though, *T* ^2^ behavior of resistivity has been reported for several 3D Fermi liquid, it is rarely observed in 2D Fermi liquid systems. After creating a lateral magnetic superlattice, *T* ^2^-dependent resistivity due to electron-electron umklapp scattering has been observed in 2D electron gas at GaAs/AlGaAs heterointerface^25, 26^. Although the relaxation time (*τ*) of quasiparticle for a Fermi liquid system depends on the dimensionality, the temperature dependence of relaxation time in electronic transport can differ from that of quasiparticle. For example, *τ* ^−1^ ∝ *T* ^2^ in the 3D case whereas *τ* ^−1^ ∝ *T* ^2^ ln(*E*_*F*_/*k*_*B*_ *T*) in the 2D case, where *E*_*F*_ is the Fermi energy^27^. However, the resistivity is proportional to *T* ^2^ in 3D as well as 2D systems^27^.

### Magnetoresistance and weak anti-localization (WAL) effect in Bi_1.5_Sb_0.5_Te_1.7_Se_1.3_ single crystals

**Figure 2:**
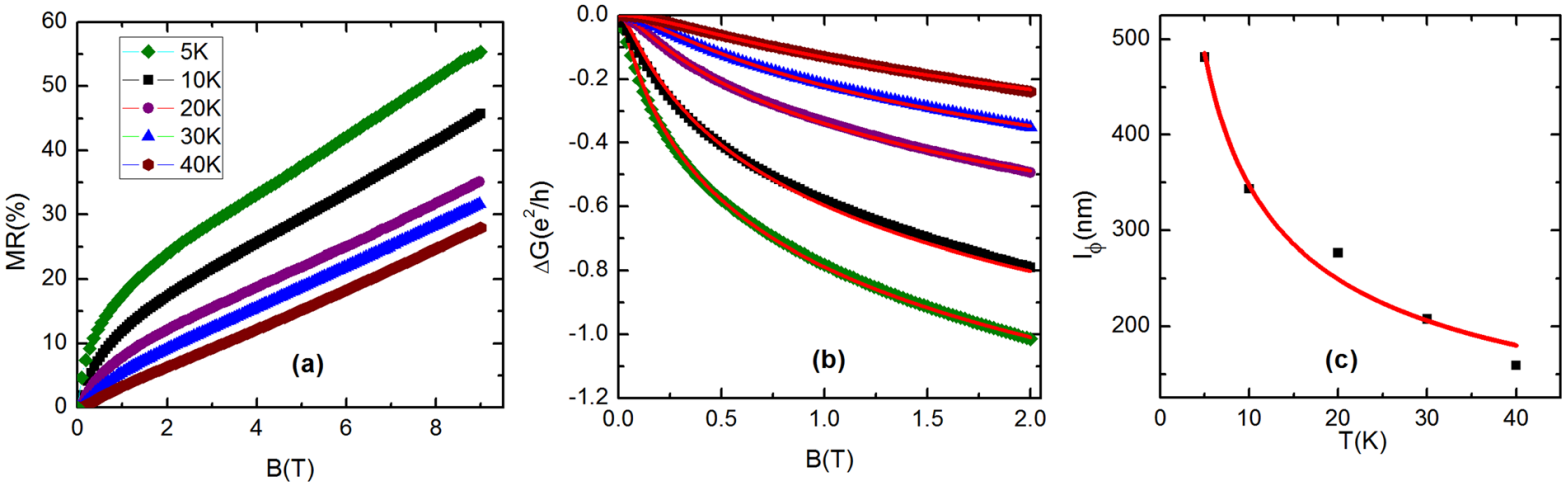
(**a**) Magnetic field dependence of the magnetoresistance (MR) for the S2 crystal at different representative temperatures between 5 and 40 K. (**b**) Field dependence of the surface conductance (*G*), which has been calculated using the expression, $G=\frac{{\rho }_{xx}}{{\rho }_{xx}^{2}+{\rho }_{xy}^{2}}t$. Here, *ρ*_*xy*_ is the Hall resistivity and *t* is the thickness of the sample. Solid lines are the fit to the experimental data with HLN equation (mentioned in the text). (**c**) Dephasing length is plotted against temperature, which follows the expected *T* ^−0.5^ dependence, as shown by the red line.

The magnetoresistance (MR) defined as [*ρ*_*xx*_(*B*) − *ρ*_*xx*_(0)]/*ρ*_*xx*_(0) has been measured in the field (*B*) range 0–9 T. The field dependence of MR at different temperatures is shown in Fig. 2(a) for the S2 crystal as a representative. MR increases almost linearly in the high-field region. The nonlinearity in MR at low field is due to the lifting of weak anti-localization effect under application of magnetic field^17, 28^. Magnetic field breaks the time reversal symmetry and opens a gap in the surface Dirac cone state. Hence, the rate of change of magnetoresistance, which is maximum in the limit *B* → 0, decreases with the increase in field strength. The WAL effect is solely a surface property and it’s signature in MR has been observed in thin films of almost all 3D TIs like Bi_2_Se_3_ Bi_2_Te_3_, Sb_2_Te_3_^28, 29^. As the bulk state of these materials is metallic in nature due to the uncontrolled doping, one has to reduce the bulk contribution to conductivity by thinning the sample to a nanometer scale to observe surface dominated phenomena like WAL effect. In such case, the charge conduction through surface channel dominates over the bulk and one can use the well known Hikami-Larkin-Nagaoka (HLN) formula to describe the WAL effect^30^,

1 $${\rm{\Delta }}G(B)=-\frac{\alpha {e}^{2}}{2{\pi }^{2}\hslash }[\psi (\frac{1}{2}+\frac{{B}_{\varphi }}{B})-\,\mathrm{ln}(\frac{{B}_{\varphi }}{B})].$$

Here, the magneto-conductance (Δ*G*) is defined as Δ*G* = [*G*(*B*) − *G*(0)], *G* is the surface channel conductance, the parameter *α* represents the number of conduction channels (i.e., *α* = 0.5 for each conducting channel), ${B}_{\varphi }=\frac{\hslash }{4e{l}_{\varphi }^{2}}$ and *l*_*ϕ*_ is the dephasing length. On the other hand, if the bulk contribution to conductivity can be reduced significantly by some other means - one of such is by making the bulk highly insulating, then the WAL effect can be observed even in a thick sample. BSTS has been designed to hold very poor bulk conductivity due to a gap in the density of states in the bulk electronic band, as discussed in the introduction section of our manuscript. The charge transport in these materials shows semiconducting-like behavior, and the surface is almost electronically isolated from the bulk at low-temperature. As a consequence, one can easily observe the WAL effect and fit the data with 2D HLN formula in transport studies on 3D single crystals of these materials^12, 16^.

In the studied samples, the quadratic field dependence of magnetoresistance from the bulk electronic state is absent at low temperature. Thus, MR in the low-temperature region originates from the conducting surface state only. As *ρ*_*xx*_ is a tensor quantity in presence of magnetic field, *σ*_*t*_ has been calculated using the relation *G*/*t* = *σ*_*t*_ = *ρ*_*xx*_/$[{\rho }_{xx}^{2}+{\rho }_{xy}^{2}]$, where *ρ*_*xy*_ is the Hall resistivity. We have fitted the magneto-conductivity data with the above mentioned HLN equation for fields below 2 T and shown in Fig. 2(b). The excellent fitting between experimental data and theoretical expression confirms that the low-temperature MR is due to the weak anti-localization effect. As shown in Fig. 2(c), *l*_*ϕ*_ reduces drastically with increasing temperature, following the theoretically predicted temperature dependence, *l*_*ϕ*_ ∝ *T* ^−0.5^ ^31–34^. We have also extracted the value of the parameter *α*, which is ~1 at 5 K and 10 K but it gradually decreases with increase in temperature and drops down to 0.8 at 40 K. The value of *α* ~ 1 implies that two conducting channels, the top and bottom surfaces of the single crystal, are taking part in charge conduction at low temperature. These observations further support that the low-temperature transport is dominated by the surface state. At high temperature, as a significant fraction of the total current passes through the bulk, the value of *α* decreases from 1.

### The Hall resistivity and aging effect

**Figure 3:**
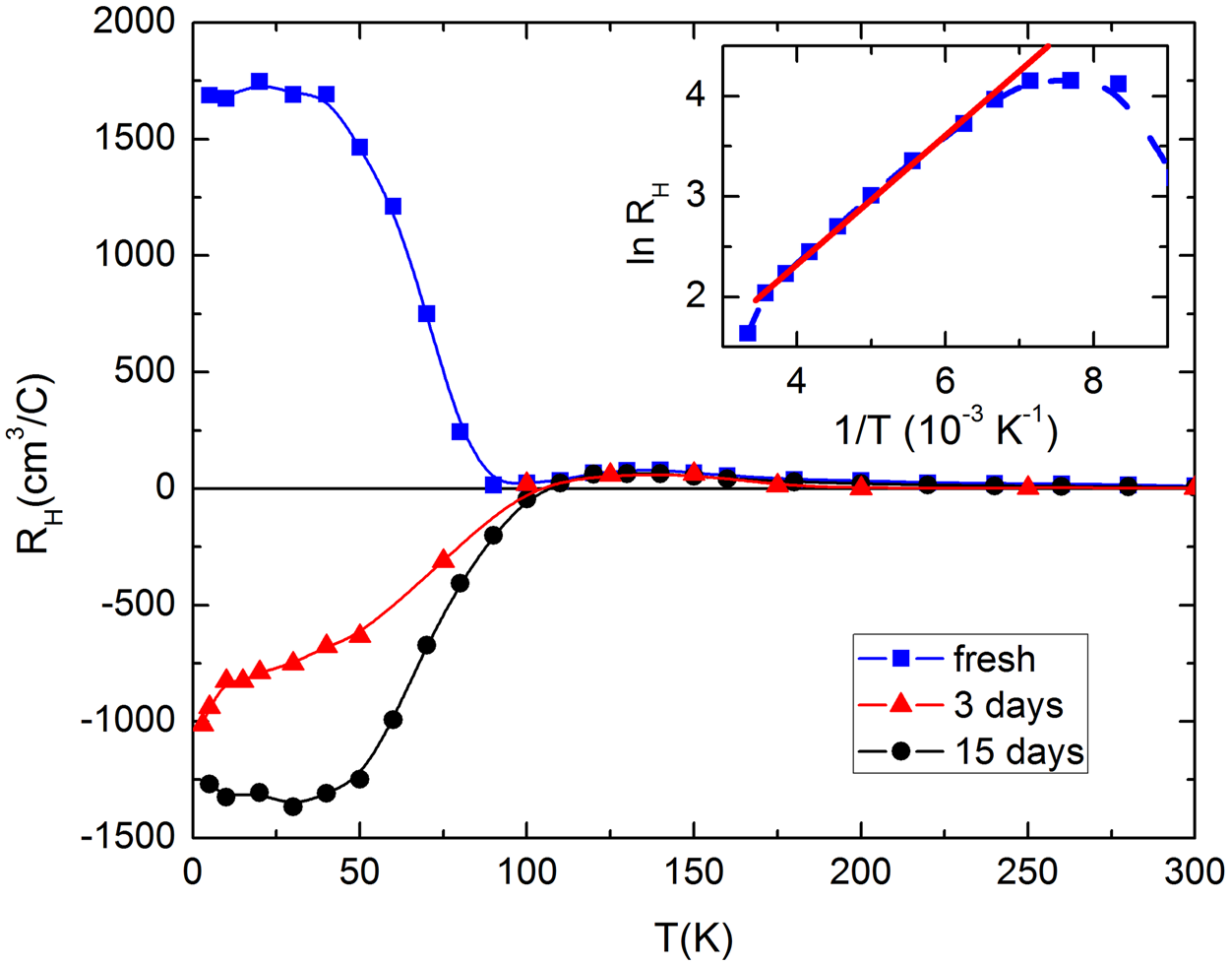
Temperature dependence of the low-field Hall coefficient (*R*_*H*_) for Bi_1.5_Sb_0.5_Te_1.7_Se_1.3_ single crystal (S2) at different ages. The blue squares are the *R*_*H*_ data for the freshly cleaved S2 crystal. The red triangle and black solid circle represent *R*_*H*_ data for the same S2 sample after being exposed to air for 3 days and 15 days, respectively. Inset shows ln(*R*_*H*_) vs 1/*T* plot for freshly cleaved S2 crystal. The solid line shows the linear behavior of ln(*R*_*H*_) at high temperatures above 160 K.

The temperature dependence of the Hall coefficient (*R*_*H*_) for several freshly prepared and aged crystals has been measured. For these crystals, *R*_*H*_ is positive and shows activation behavior at high temperature. The positive sign of *R*_*H*_ implies that holes are the majority carriers at high temperature. *R*_*H*_(*T*) for both freshly prepared and aged S2 crystals is shown in Fig. 3 as representative. The magnetic field dependence of the Hall resistivity for the freshly cleaved crystal is shown in Supplementary Fig. S3. As the Fermi energy of BSTS lies in the gap between the conduction and valence band of the bulk, *R*_*H*_ exhibits activation behavior^23^. The value of activation energy deduced from the Hall data is about 39 meV which is very close to that calculated from resistivity. Valance band being closer to the Fermi level, *p*-type carrier dominates the charge conduction in the bulk. The observed behavior of *R*_*H*_(*T*) in the range 125–300 K is qualitative similar to earlier reports on samples close to optimum composition^10, 11, 19^. The freshly cleaved samples have *p*-type surface state. But, the aged samples show *n*-type carrier at low temperature as indicated by the sign of *R*_*H*_ below ~100 K. It has been observed that exposure to atmosphere causes electron doping on the surface, as a result, the Fermi energy shifts above the Dirac point^10^. Thus, the *R*_*H*_ for the crystals those were exposed to air for longer time, starts to decrease sharply below 125 K and becomes negative at low temperature. This type of aging effect is quite common in topological insulator. In the subsequent discussion, we will show that the aging effect of topological surface state can be observed from the magnetic measurements on these materials.

### Singular paramagnetic response in the magnetic susceptibility

The low-energy physics of the surface state for a three-dimensional topological insulator can be described by the Dirac type effective Hamiltonian, H_*sur*_(*k*_*x*_, *k*_*y*_) = *ħv*_*F*_(*σ* ^*x*^ *k*_*y*_ − *σ* ^*y*^ *k*_*x*_), where *v*_*F*_ is the Fermi velocity^35, 36^. This implies that the spin ($\overrightarrow{\sigma }$) and momentum wave vector $(\overrightarrow{k})$ of low-energy quasi particle excitations are always perpendicular to each other for the eigenstate of the Hamiltonian, known as “spin-momentum locking”. If we define a helicity operator, $\hat{h}=(1/k)\hat{z}$. $(\overrightarrow{k}\times \overrightarrow{\sigma })$ for the spin texture on circular constant energy contour of the surface state Dirac cones, it is left-handed for the upper Dirac cone and right-handed for the lower Dirac cone. However, at the Dirac node, where the two bands with opposite spin helicity touch each other, the spins are free to align along the external magnetic field due to the singularity in spin orientation. This intrinsic paramagnetic contribution to the magnetic moment of the system is expected to reflect in the susceptibility curve *χ*(*B*). As the number of electrons close to the Dirac point is very small in amount as compared to the number of states contributing to diamagnetic signal, it is expected that the paramagnetic contribution will be very small. This predicts a low-field singular paramagnetic peak in the susceptibility curve *χ*(*B*).

Experimental discovery of this singular peak in *χ* has been reported for the family of 3D topological insulators and identified as the fingerprint for the helical spin texture of the Dirac fermions for the surface state^37–39^. *χ* shows linear-in-field decay from its zero-field value^37–39^, which has also been established theoretically^37^. At *T* = 0 and the sample’s native chemical potential (*μ* = 0), this areal (sheet) paramagnetic susceptibility due to the electrons close to the Dirac point has the form,

2 $${\chi }_{A}\cong \frac{{\mu }_{0}}{4{\pi }^{2}}[\frac{{(g{\mu }_{B})}^{2}}{\hslash {v}_{F}}{\rm{\Lambda }}-\frac{\mathrm{2(}g{\mu }_{B}{)}^{3}}{{(\hslash {v}_{F})}^{2}}|B|],$$

where *g* and *μ*_*B*_ are the Landé *g*-factor and Bohr magneton, respectively. Λ is the effective size of the momentum space, which is responsible for the singular behavior of *χ*. The areal susceptibility (*χ*_*A*_) is related to the experimental susceptibility *χ* through some multiplicative constants, $\chi ={\chi }_{0}+{\chi }_{A}\frac{x}{{L}_{z}}$. Here, *χ*_0_, *L*_*z*_ and *x* are the diamagnetic susceptibility of the background, thickness of the sample and the fraction of the surface contributing to the singular part of the free energy, respectively^37^. As the height of the peak in *χ* is determined by Λ, depending on the details of the bulk band, the peak height can vary from system to system within the family of 3D topological insulators^37^. However, the nature of the topological response determined by the cuspiness, the linear-in-field decay, robustness against temperature, etc., are universal to the entire family of 3D topological insulators^37^.

**Figure 4:**
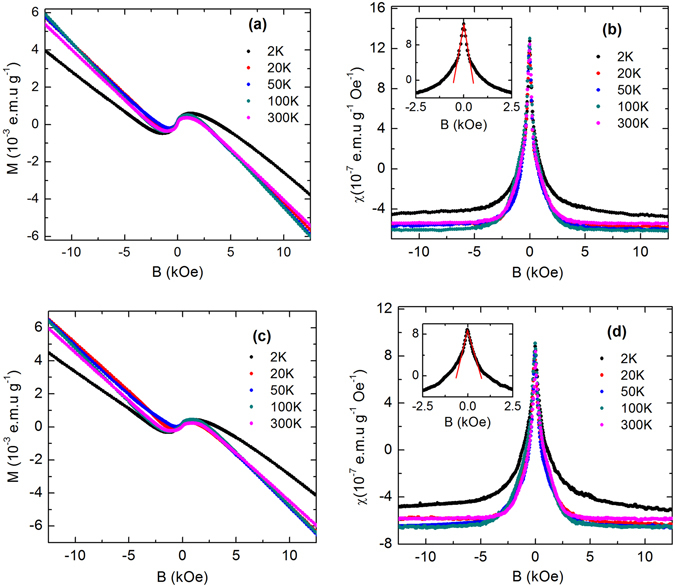
(**a**) Magnetization (*M*) versus *B* for the freshly cleaved single crystal (S2) of Bi_1.5_Sb_0.5_Te_1.7_Se_1.3_ at several representative temperatures from 2 to 300 K, (**b**) Susceptibility $(\chi =\frac{dM}{dB})$ as a function of *B*, calculated by taking the first-order derivative of magnetization. Inset shows the linear-in-field decay of *χ* from its zero-field value at a representative temperature 2 K for the freshly cleaved sample. (**c**) Magnetization for the same piece of S2 crystal of Bi_1.5_Sb_0.5_Te_1.7_Se_1.3_, which was kept in air for three days after the first measurement. (**d**) Susceptibility as a function of *B* for the aged S2 crystal at several representative temperatures. Inset shows the linear-in-field decay of *χ* at 2 K.

Figure 4(a) shows magnetization (*M*) of the freshly cleaved single crystal (S2) as a function of magnetic field, at several representative temperatures between 2 and 300 K. *M* shows diamagnetic behavior over a wide field range except at low fields, where a sharp paramagnetic upturn emerges. This is clearly visible from *χ* versus *B* plot, as shown in Fig. 4(b). The experimental value of *χ* has been obtained after taking the first-order derivative of *M*(*B*) with respect to *B*. A cusp-like paramagnetic response in *χ*(*B*) sharply rises over the diamagnetic background in a narrow field range ~±1.5 kOe. Irrespective of temperature, the height of the peak from the diamagnetic background remains almost same. This singular peak shows no sign of thermal rounding up to the highest measuring temperature 300 K. In this context, we would like to mention that the magnetization data for the starting elements Bi, Sb, Se and Te, used for sample preparation show the usual diamagnetic behavior [See Supplementary Fig. S4]. None of the elements exhibits paramagnetic like behavior due to the magnetic impurity. We have also measured the magnetic moment of blank sample holder, and compared the background and sample data [See Supplementary Fig. S5]. The magnetic moment of the blank sample holder in Fig. S5(a) is found to be 10^3^ times smaller than the measured magnetic data of our BSTS sample [Fig. S5(b)] and it does not show any anomaly in the low-field region. Hence, any spurious contribution from the holder to the BSTS data is insignificant. Furthermore, the paramagnetic signal in BSTS is observed to increase when the surface area was increased by cleaving a single crystal of given mass and volume into thin slices (See Supplementary Fig. S6). For any magnetic impurity, however, one cannot enhance *χ* by cleaving the single crystal. These observations clearly demonstrate that, similar to Bi_2_Se_3_, Sb_2_Te_3_, Bi_2_Te_3_^37, 38^ and ZrTe_5_^39^, the paramagnetic response in the present system is due to the helical spin texture of the 2D Dirac fermion on the surface.

Inset of Fig. 4(b) shows the linear decay of *χ* with field from its zero-field value at a representative temperature 2 K, as predicted theoretically (Eq. 2). Thus one can show, $\frac{d\chi }{dB}=\frac{{\mu }_{0}}{2{\pi }^{2}}\frac{{(g{\mu }_{B})}^{3}}{{(\hslash {v}_{F})}^{2}}\times \frac{x}{{L}_{z}}$. Using the sample thickness, *L*_*z*_ ~ 0.1 mm and the reported value of *v*_*F*_ ~ 4.5 × 10^5^ m/s^14^, we deduce a relation between *g*-factor and *x*, $g \sim \frac{35}{{x}^{1/3}}$. As *x* is always less than 1, the value of *g*-factor appears to be greater than 35. This implies that the spin-orbit coupling in the present compound is very strong. The value of *g*-factor has been reported to vary over a wide range from 20 to 76 for this class of compounds^10, 37, 40–42^, which is consistent with our experimental result. When the surface is exposed to air for long time, the surface reconstruction and the formation of 2D electron gas occur due to the bending of bulk band^37^. As a result, the peak height has been observed to reduce with time^37, 39^. To probe the aging effect in BSTS, the magnetization measurements were done on the same piece of S2 single crystal after 3 days of exposure to air and shown in Fig. 4(c). Although the nature of the peak along with the diamagnetic background, as shown in Fig. 4(d), remain unchanged, a significant drop (~25%) is observed in the height, reflecting the expected aging effect in the present sample. Inset of Fig. 4(d) shows the linear decay of *χ* with field at 2 K for the aged sample.

## Discussions

We observe a crossover from bulk dominated insulating to surface dominated metallic behavior in the temperature dependence of resistivity for Bi_1.5_Sb_0.5_Te_1.7_Se_1.3_ single crystals. The competition between surface and bulk conduction appears to be very sensitive to the sample preparation. A rare quadratic temperature dependence of resistivity for the metallic surface conduction has been observed for samples with large bulk resistivity. This highly decoupled metallic surface, which was remained elusive in earlier studies, is an essential criterion for the use of spin-momentum locked topological state in electronic application and basic research. In addition, we observe a sharp cusp-like paramagnetic peak at zero field in magnetic susceptibility. This temperature insensitive anomalous magnetic response, has been attributed to the helical spin texture of 2D Dirac fermion on the surface of 3D topological insulators. The surface state reconstruction with time due to the doping from air, known as aging effect for 3D topological insulators, is clearly reflected from the age dependent reduction of the susceptibility peak.

## Method

Single crystals of Bi_1.5_Sb_0.5_Te_1.7_Se_1.3_ were prepared by self-flux method using high purity elements (5 N) of Bi, Sb, Te and Se in a stoichiometric ratio 1.5:0.5:1.7:1.3, respectively. Two slightly different techniques were adopted to prepare these crystals. In both the cases, sample handling was done inside a glove box in argon gas atmosphere. Method I: Single crystal S3 was prepared in this method. Stoichiometric mixture of Bi, Sb, Te and Se granules was vacuumed sealed in a quartz tube and then heated at the rate of 70 °C/h to 850 °C and kept at that temperature for 48 h for diffusion process. The sample was cooled over a period of 60 h to 550 °C and kept at that temperature for 96 h before furnace cooling. Method II: Some minor modifications were done over method I to prepare single crystals S1 and S2. The stoichiometric mixture of elements was pelletised before the vacuum sealing. In this method, at the final stage (of method I) the sample was slowly cooled from 550 °C to room temperature at the rate of 10°/h instead of furnace cooling. The local temperature gradient, which sustains due to the slow cooling process, in the vertically placed tube helps in stacking of chalcogenite layer and growing large single crystalline flake which can be easily cleaved using a razor. The typical thickness of the crystals used for electrical transport and magnetic measurements is ~80 to 110 *μ*m. In-plane electrical resistivity and the Hall coefficient were measured by four-probe technique in a physical property measurement system (Quantum Design). Magnetization measurement was performed in SQUID-VSM (Quantum Design). Before magnetization measurement of BSTS, the system was counterchecked with elements such as Bi, Sb, Te, and Se those were used to prepare Bi_1.5_Sb_0.5_Te_1.7_Se_1.3_ crystals and also with the standard palladium sample. The details are discussed in the supplementary section along with sample characterization.

## Electronic supplementary material

Supplementary Information
