# Supplementary material for: Prominent metallic surface conduction and the singular magnetic response of topological Dirac fermion in three-dimensional topological insulator Bi1.5Sb0.5Te1.7Se1.3
Source: Sci Rep. 2017 Jul 7;7:4883. doi: 10.1038/s41598-017-05164-9 (PMC5501823; doi:10.1038/s41598-017-05164-9)
Supplement: Supplementary file 1 — Supplementary Information [file 41598_2017_5164_MOESM1_ESM.pdf]

SI for "Prominent metallic surface conduction and the singular magnetic response of topological Dirac fermion in three-dimensional topological insulator $Bi_{1.5}Sb_{0.5}Te_{1.7}Se_{1.3}$"

Prithwish Dutta[1,2], Arnab Pariari[1], and Prabhat Mandal[1*]
[1]*Saha Institute of Nuclear Physics, HBNI, 1/AF Bidhannagar, Calcutta 700 064, India and*
[2]*Goverment General Degree College, Singur, Hooghly 712409, India*

[*] prabhat.mandal@saha.ac.in

A. Sample characterization using powder x-ray diffraction and energy dispersive x-ray techniques

The phase purity of $Bi_{1.5}Sb_{0.5}Te_{1.7}Se_{1.3}$ (BSTS) single crystals was checked by high resolution powder x-ray diffraction (XRD) method with Cu K_α radiation in a Rigaku x-ray diffractometer (TTRAX II). Within the resolution of XRD, we have not observed any peak due to impurity phase. Fig. S1(a) shows the diffraction pattern for powdered crystal prepared by method II as a representative. All the peaks in the diffraction pattern can be fitted well with the hexagonal unit cell of space group symmetry R$\bar{3}$m using the Rietveld refinement. The calculated lattice parameters are a_{hex}=4.2840 Å and c_{hex}=29.871 Å. Inset of Fig. S1(a) shows (1 0 7) and (0 0 12) peaks. The presence of these peaks are very important for the ordering of chalcogen layer. We have also performed the x-ray diffraction on a single flake as shown in Fig. S1(b). The Bragg peaks, which are integer multiple of (003) only are observed, indicating good c-axis orientation of the single crystal. The (0 0 12) peak is also very clearly observed in the diffraction pattern. Energy dispersive x-ray (EDX) (Quanta 200, FEG scanning electron microscope) analysis was used to determine the chemical composition of the sample. Identical chemical composition at different positions on the surface of the single flake and for different flakes suggest that the samples are chemically homogeneous. Fig. S2 shows the typical EDX spectrum of one such BSTS single crystal. The chemical composition determined using EDX is close that of nominal composition, at least within the limits of the SEM-EDX analysis.

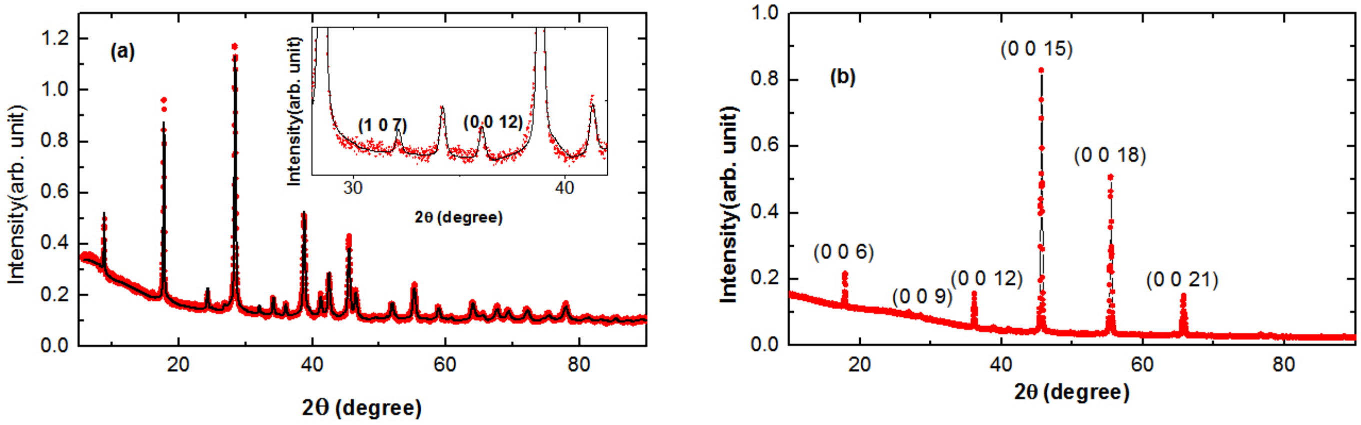

FIG.S 1. (a) X-ray diffraction pattern of powdered sample for $Bi_{1.5}Sb_{0.5}Te_{1.7}Se_{1.3}$ single crystals (S2). Red circles are experimental data and the black continuous line corresponds to Rietveld refinement of the diffraction pattern. The Bragg peaks (1 0 7) and (0 0 12) are shown in the inset. (b) X-ray diffraction pattern for a single flake shows less number of Bragg peaks than the powdered sample with peak index integer multiple of (003).

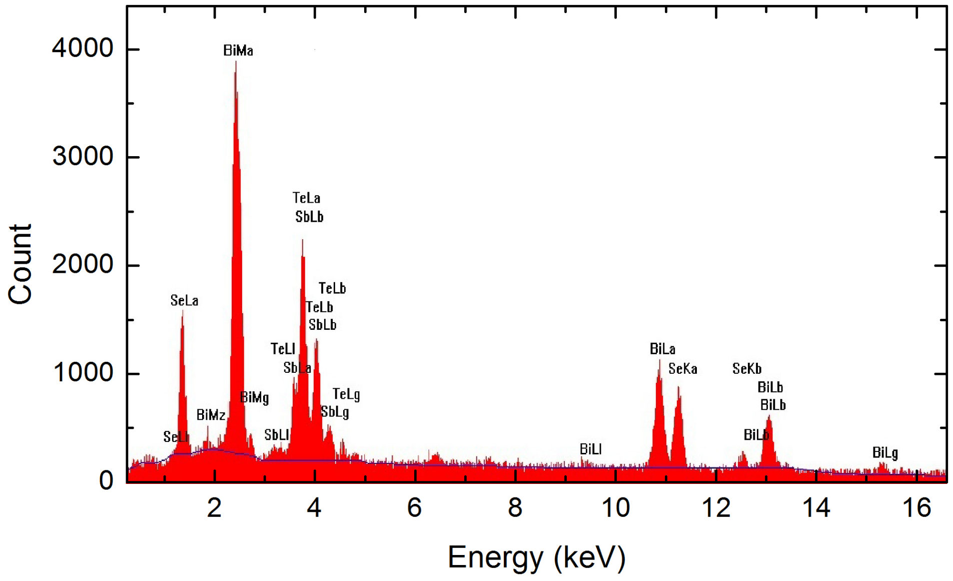

FIG.S 2. Energy dispersive x-ray spectra for $Bi_{1.5}Sb_{0.5}Te_{1.7}Se_{1.3}$ single crystal.

B. Field dependence of the Hall resistivity

The magnetic field dependence of the Hall resistivity (ρ_{xy}) of the freshly cleaved S2 crystal is plotted in Fig. S3 at some representative temperatures between 5 and 200 K. The slope of ρ_{xy} *vs* B plot at low field, provides the value of Hall coefficient (R_H) at that temperature and is discussed in the manuscript. ρ_{xy} is positive and increases almost linearly with field from room temperature down to $\sim$ 120 K. This behavior of R_H indicates that p-type charge carrier dominates the conductivity of the bulk at high temperature. As the temperature decreases further, the nonlinearity in ρ_{xy} *vs* B starts to appear because the surface contribution becomes significant due to the exponential decrease in bulk carrier.

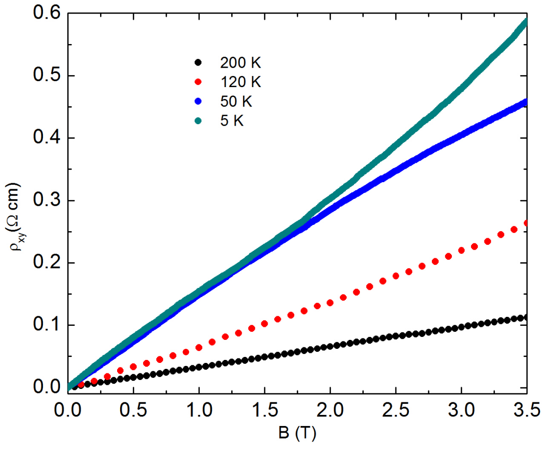

FIG.S 3. Magnetic field dependence of the Hall resistivity (ρ_{xy}) at some representative temperatures. ρ_{xy} at 120 K and 200 K has been multiplied by factor 10.

C. Magnetic measurements of standard samples

We have also measured the field response of magnetic moment of elemental bismuth, antimony, tellurium, selenium, and palladium and the empty sample holder of our instrument, prior to BSTS single crystal. Fig. S4(a) shows that

the linear diamagnetic moment of bismuth at 2 and 100 K passes through the origin. This is more clear from Fig. S4(b), which shows magnetic field dependence of differential susceptibility ($\chi=\frac{dM}{dB}$). Similar to bismuth, selenium and tellurium also show the expected diamagnetic behaviour irrespective of temperature and shown in Fig. S4(c) and Fig. S4(e), respectively. The susceptibility, χ, derived from the data is shown in Fig. S4(d) and Fig. S4(f). χ does not show any peak at low field. In case of antimony, however, a slight deviation from the linear field dependence of magnetization is observed near zero field and at low temperature, which vanishes at higher temperature [Fig. S4(g)]. The differential susceptibility ($\chi=\frac{dM}{dB}$) is plotted in Fig. S4(h) and unlike BSTS, it is a broad diamagnetic cusp near zero field. The observed behavior of magnetization in these elements is very similar to earlier report [1]. The overall magnetic behaviour implies the absence of any paramagnetic impurity in the starting elements.

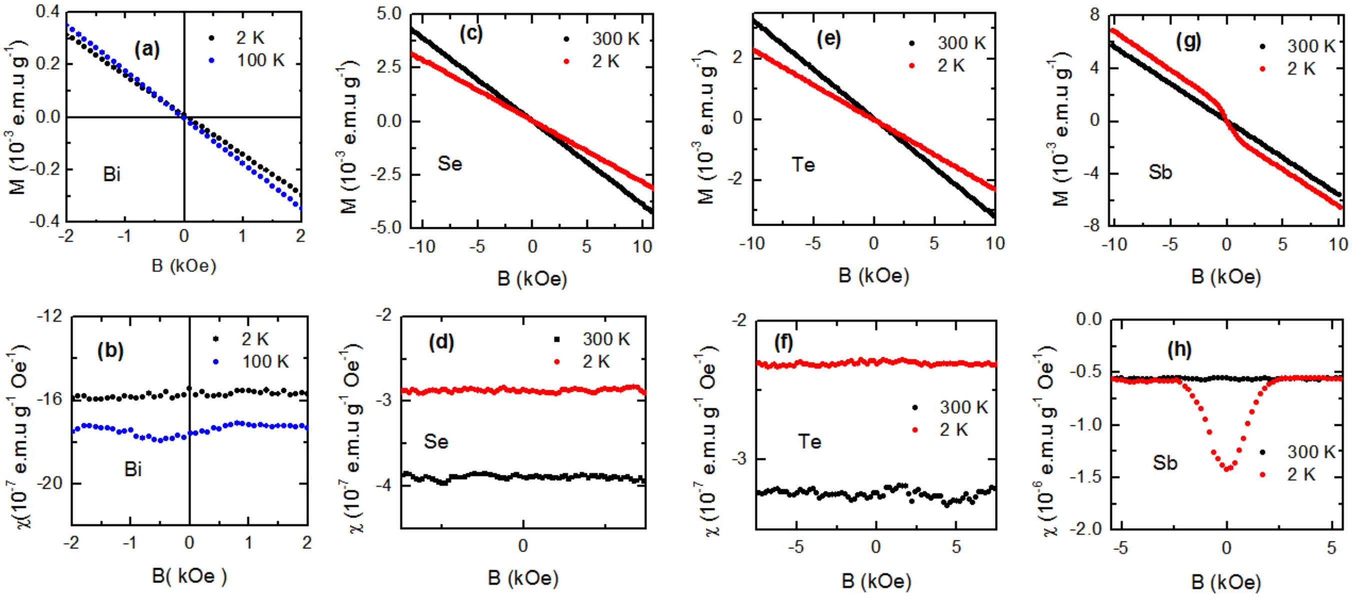

FIG.S 4. (a), (c), (e) and (d) Magnetization of bismuth, selenium, tellurium, and antimony respectively, which have been used to prepare the single crystal of $Bi_{1.5}Sb_{0.5}Te_{1.7}Se_{1.3}$. (e), (d), (f) and (g) The differential susceptibility ($\chi=\frac{dM}{dB}$) for the respective elements obtained after taking the numerical derivative of the magnetization with respect to external magnetic field.

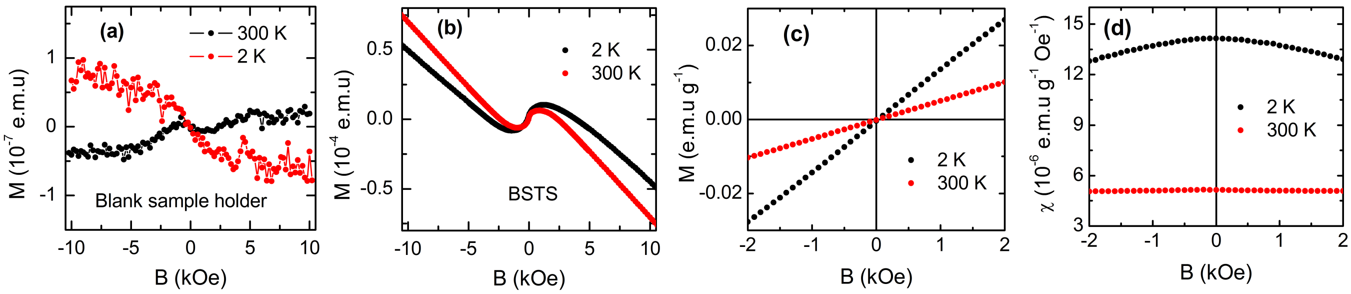

FIG.S 5. (a) Magnetic moment for the blank sample holder. (b) The raw data of magnetic moment for BSTS. (c) Magnetization of standard paramagnetic palladium sample at 2 and 300 K. (d) Susceptibility ($\chi=\frac{dM}{dB}$) at the corresponding temperatures.

The magnetic moment of the blank sample holder in Fig. S5(a), is 10^3 times smaller than the raw data of measured magnetic moment of BSTS, as shown in Fig. S5(b). Also, the magnetic moment of the sample holder does not show any anomalous behavior in the low-field region. Hence, any contribution from the sample holder to the experimental data will be insignificant. This confirms that the singular paramagnetic susceptibility in BSTS single crystal is not due to any spurious response in our system. Fig. S5(c) shows the expected magnetic behaviour of paramagnetic palladium sample, provided by Quantum Design. The low-field susceptibility of palladium at 2 K and room temperature is shown in Fig. S5(d). The nonlinear behaviour of χ at low field and the broad zero field peak at 2 K are

completely suppressed with increasing temperature. This behavior is entirely different from the singular, robust and linear low-field paramagnetic response of the topological surface state in the present sample.

D. Magnetization of $Bi_{1.5}Sb_{0.5}Te_{1.7}Se_{1.3}$ single crystal (S2) before cleaving

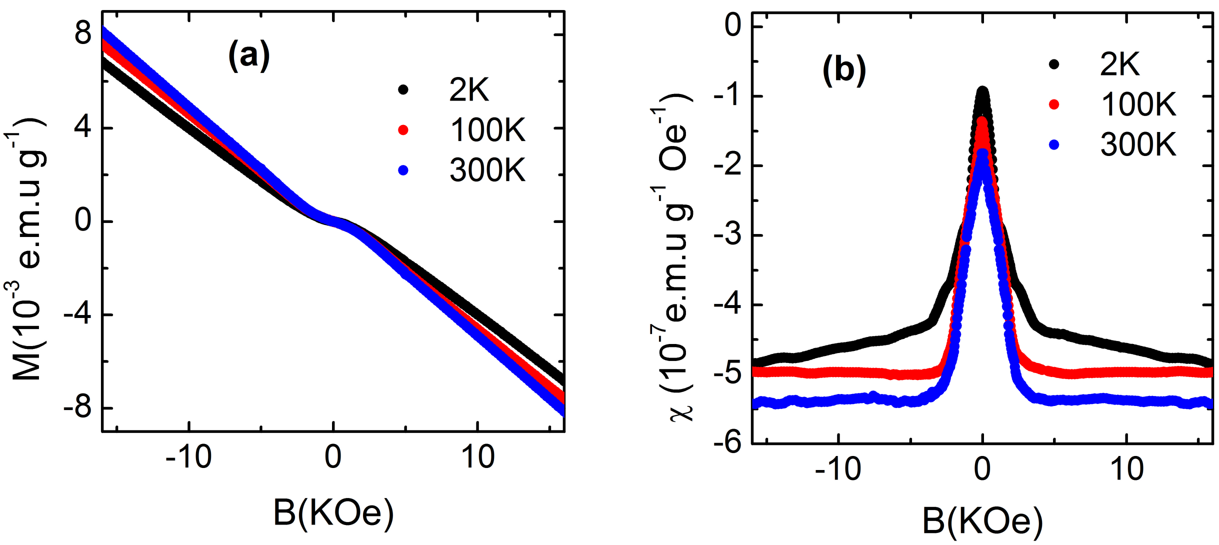

FIG.S 6. (a) Magnetization (M) vs B plot for single crystal (S2) of $Bi_{1.5}Sb_{0.5}Te_{1.7}Se_{1.3}$ before cleaving into thin pieces at representative temperatures 2, 100 and 300 K, (b) Susceptibility ($\chi = \frac{dM}{dB}$) as a function of B, calculated by taking the first-order derivative of magnetization.

Fig. S6 shows the paramagnetic response of $Bi_{1.5}Sb_{0.5}Te_{1.7}Se_{1.3}$ single crystal (S2) before cleaving into thin pieces, i.e., before increasing the surface area keeping the mass and volume fixed. It is evident from the figures that the paramagnetic upturn behavior in magnetization and its associated singular peak in susceptibility is nearly four times lower as compared to that observed in cleaved samples in Figure 4 (a) and 4 (b). This can also be consider as an indication that the singular paramagnetic peak in susceptibility is related to surface state, not due to magnetic impurities.

References

1. Zhao, L. *et al.* Singular robust room-temperature spin response from topological Dirac fermions. *Nature Material* **13**, 580 (2014).
